# Radiofrequency-Induced Thermal Modulation Reduces Senescence-Induced Collagen Fiber Degradation in Facial Ligaments of Animal Models

**DOI:** 10.3390/cells14221757

**Published:** 2025-11-10

**Authors:** Seyeon Oh, Hyoung Moon Kim, Gwahn Woo Cheon, Geebum Kim, Kuk Hui Son, Kyunghee Byun

**Affiliations:** 1 Functional Cellular Networks Laboratory, Lee Gil Ya Cancer and Diabetes Institute, Gachon University, Incheon 21999, Republic of Korea; 2 LIBON Inc., Incheon 22006, Republic of Korea; 3 Department of Anatomy & Cell Biology, College of Medicine, Gachon University, Incheon 21936, Republic of Korea; 4 Maylin Clinic, Goyang 10391, Republic of Korea; 5 Maylin Clinic, Pangyo 13529, Republic of Korea; 6 Misogain Dermatology Clinic, Gimpo 10108, Republic of Korea; 7 Department of Thoracic and Cardiovascular Surgery, Gil Medical Center, College of Medicine, Gachon University, Incheon 21565, Republic of Korea; 8 Department of Health Sciences and Technology, Gachon Advanced Institute for Health & Sciences and Technology (GAIHST), Gachon University, Incheon 21999, Republic of Korea

**Keywords:** radiofrequency, facial ligament, HSP70, senescence

## Abstract

**Highlights:**

**What are the main findings?**

- Radiofrequency (RF) increased HSP70 expression and enhanced HSP70-IKKγ binding, thereby reducing IκBα phosphorylation and NF-κB activation in senescent fibroblasts and facial ligaments.
- RF restored the collagen type I/III ratio and collagen fiber density in senescent facial ligaments, with more favorable molecular responses observed at 42 W than at 73 W.

**What are the implications of the main findings?**

- These findings suggest that HSP70 plays a central role in mediating RF-induced molecular remodeling during ligament senescence.
- RF provides preclinical evidence of a temperature-dependent mechanism that mitigates collagen degradation and supports extracellular matrix homeostasis in aging connective tissues.

**Abstract:**

Age-related changes in facial ligaments contribute to altered facial shape and soft tissue descent. Radiofrequency (RF) has been utilized for skin rejuvenation by promoting collagen fiber contraction and synthesis through increased expression of heat shock proteins (HSPs). The primary component of ligamentous collagen fibers undergoes structural modifications with age, exhibiting increased fragmentation and a reduced collagen type I/III ratio. This study aimed to investigate whether RF irradiation alleviates senescence-related changes in facial ligaments through HSP70-mediated molecular remodeling using a UV-induced photoaging rat model. In senescent fibroblasts, RF enhanced the interaction between HSP70 and IκBα kinase (IKK)γ while reducing IκBα phosphorylation, which was associated with decreased nuclear factor-kappa B (NF-κB) activation. These RF-mediated changes were attenuated by an HSP70 inhibitor, suggesting that RF reduces NF-κB activity via HSP70 modulation. RF also suppressed expression levels of matrix metalloproteinases and SMAD7 in senescent fibroblasts. Consistent with in vitro findings, RF increased the interaction between HSP70 and IKKγ while decreasing IκBα phosphorylation and NF-κB activity in the UV-induced photoaging (senescent) facial ligaments of rat models. Furthermore, RF enhanced the collagen type I/III ratio and increased collagen fiber density within the ligaments. Scanning electron microscopy revealed that RF irradiation increased collagen fiber bundle diameter and enhanced the helical structure of those fibers. Overall, RF mitigates senescence-related changes in facial ligaments through HSP70 modulation. Considering that facial ligament laxity contributes to soft tissue descent, facial ligament-targeting approaches may promote a more youthful facial structure. RF demonstrates the possibility in reducing senescence-associated changes within facial ligaments.

## 1. Introduction

During the facial aging process, changes occur within all layers of facial structures, including collagen-containing tissue and facial fat [1]. Collagen-containing facial tissues (e.g., dermis, fibrous septa, superficial muscular aponeurotic system, and retaining ligaments) exhibit stretching, attenuation, and fragmentation with age [1]. Furthermore, facial fat tissue undergoes migration, leading to soft tissue descent and the loss of a youthful facial shape [2,3]. Facial ligaments function as osteocutaneous connections, anchoring skin to the underlying bone [4]. Age-related enhancement of facial ligament laxity may contribute to facial soft tissue descent [5]. Reduced structural support from facial ligaments may also facilitate the formation of jowls, nasolabial folds, and marionette lines [6].

Collagen fibers—primary structural proteins of the extracellular matrix (ECM) in the dermis—maintain a tightly packed and organized network in young skin [7]. During aging, collagen fibers undergo structural changes, becoming fragmented and unevenly distributed; their quantity also decreases [8]. This degradation is primarily mediated by matrix metalloproteinases (MMPs), a group of endopeptidases [9]. Age-related accumulation of reactive oxygen species promotes inflammation-associated factors, including activator protein-1 and nuclear factor-kappa B (NF-κB) [9]. NF-κB serves as a key regulator that enhances the transcription of MMP1 and MMP3, leading to ECM degradation [9].

Collagen fibers also constitute primary structural components of ligaments and tendons [10]. During aging, ligaments undergo changes including a decrease in collagen fibril size, as well as increases in fragmented and disorganized collagen fibers [10,11]. Type I collagen is the predominant collagen type, comprising 95% of the total collagen content in tendons [12]; it plays a crucial role in maintaining the tensile strength of tendons [10,11,13]. Fibrillogenesis begins with the synthesis of type I collagen, followed by the sequential linear and lateral incorporation of other collagen types (e.g., III, V, XI, XII, and XIV) [14]. Whereas type I collagen is essential for maintaining mechanical stability, type III collagen, which forms thin strands, is considered juvenile collagen and primarily appears during the early wound-healing phase [15]. Increased presence of type III collagen is associated with a lower tensile modulus [12]. Greater type III collagen levels during aging are associated with decreased mechanical strength [12]. The type III/I collagen ratio increases in tendons with age and has been linked to tendon dysfunction [16,17,18].

Radiofrequency (RF) has been utilized for skin rejuvenation. RF generates heat, thereby inducing collagen fiber contraction and promoting dermal tightening [19]. This process cleaves hydrogen bonds within the collagen triple helix, resulting in fiber shortening and thickening [20]. RF stimulates wound-healing mechanisms that enhance collagen synthesis for 3–4 months after RF irradiation [20,21]. RF also increases transforming growth factor (TGF)-β expression, which promotes fibroblast activation and enhances collagen synthesis [22].

Additionally, RF plays a role in ligament healing. In an injured rat patellar ligament model, RF stimulated wound healing by increasing fibroblast activation and TGF-β expression [23]. Furthermore, RF promoted collagen synthesis by activating heat shock proteins (HSPs), including HSP70, HSP47, and HSP27 [24,25,26]. HSP70 attenuates NF-κB activation by inhibiting IκBα kinase (IKK) and facilitating IκBα degradation [27,28].

NF-κB is a heterodimeric protein comprising p65 and p50 subunits, which remain sequestered in the cytoplasm in an inactive state via binding to IκB [29]. Upon stimulation by factors such as reactive oxygen species or inflammatory cytokines, the ternary IKK complex (IKKα, IKKβ, and NEMO [IKKγ]) phosphorylates IκB at serine residues S32 and S36, leading to its degradation and subsequent NF-κB activation [30,31]. Phosphorylated IκB undergoes degradation, allowing for NF-κB release [29]. Upon liberation from IκB, NF-κB translocate to the nucleus, where it triggers the transcription of downstream target genes [29]. Additionally, HSP70 inhibits formation of the IKK complex through direct binding to IKKγ, thereby suppressing NF-κB activation [32].

NF-κB activation promotes the upregulation of SMAD7 [33]. TGF-β induces the phosphorylation of SMAD2 and SMAD3, thus enhancing collagen synthesis [34]. However, because SMAD7 inhibits the TGF-β signaling pathway [34], SMAD7 activation suppresses collagen synthesis [35].

Given that age-related changes in facial ligaments alter anatomical positioning, resulting in deviations from a youthful facial structure [2,3], methods that restore the integrity of facial ligaments represent potential esthetic approaches for reestablishing a youthful appearance.

RF reportedly increases collagen synthesis in the skin by activating HSP70 [36]. Accordingly, we hypothesized that RF could enhance collagen synthesis in facial ligaments during aging. Moreover, we speculated that RF would increase HSP70 expression, causing NF-κB suppression by promoting HSP70–IKKγ binding in conjunction with the inhibition of IκBα phosphorylation. A decline in NF-κB activity would lead to decreased MMP and SMAD7 expression, resulting in diminished collagen degradation and increased collagen accumulation in facial ligaments. We tested these hypotheses using an H_2_O_2_-induced senescent fibroblast model and ultraviolet (UV) radiation-induced animal models of senescence.

## 2. Materials and Methods

### 2.1. RF Irradiation System

The preliminary test was designed to quantitatively assess the relationship between delivered RF energy and tissue temperature, aiming to establish a safe and reproducible exposure range for subsequent experiments. The two power settings used in the in vivo ligament study (42 W and 73 W) were selected based on these preliminary results, corresponding to approximately 44.8 °C and 59 °C, which effectively induce HSP70 expression without causing thermal damage.

In this study, a monopolar RF device (Sunny; SHENB Co., Ltd., Seoul, Republic of Korea) was used as the primary exposure system for ex vivo and in vivo experiments. The device operates at a fixed frequency of 6.78 MHz [37,38,39,40,41] and delivers RF energy through a square-shaped, non-invasive application tip with an effective emitting surface area of 15 mm × 15 mm. It is equipped with an impedance monitoring system to ensure safe and stable energy delivery by measuring skin impedance during each application.

RF energy was applied using a shot duration of 1140 ms and an inter-shot interval of 400 ms, with a total of 10 shots delivered per application. The procedure was performed under five discrete power settings (42, 50, 57, 65, and 73 W), while all other parameters—including frequency (6.78 MHz), shot duration (1140 ms), inter-shot interval (400 ms), and emitting area (2.25 cm^2^; 15 × 15 mm)—were kept constant.

To characterize the energy delivered under each condition, energy fluence (J/cm^2^) was calculated using the following formula:

Fluence (J/cm^2^) = Power (W) × Pulse duration (s)/Emitting area (cm^2^)

To facilitate understanding of the following experimental models, a summary of the experimental designs for the ex vivo, in vitro, and in vivo experiments (Section 2.2, Section 2.3 and Section 2.4) is presented in Table S1.

### 2.2. Ex Vivo Study: Thermal Changes in Porcine Skin Induced by RF Irradiation

This ex vivo porcine skin test was conducted solely to characterize the physical energy distribution and temperature–power relationship of the monopolar RF device, rather than to reproduce in vivo conditions or draw any clinical conclusions.

This experiment aimed to evaluate temperature elevation in skin tissue induced by RF irradiation under various energy conditions. Excised porcine skin, which has thermal conductivity properties similar to human skin, was used to simulate dermal responses in a controlled ex vivo environment.

#### 2.2.1. Tissue Preparation and Preheating

Porcine skin tissue samples were prepared to a uniform thickness and size to ensure consistency across test conditions. To simulate thermal conditions relevant to human skin physiology, the samples were vacuum-sealed and preheated in a water bath to a temperature range of 35.5–37.0 °C. This approach was intended to provide a reference model for RF-induced thermal responses applicable to human skin. Vacuum sealing ensured uniform heat distribution and prevented water infiltration during heating. Thermal equilibrium was maintained prior to RF application to ensure comparable baseline conditions across samples.

#### 2.2.2. RF Irradiation

RF energy was delivered using the same monopolar RF device (SHENB Co., Ltd.), operating at a fixed frequency of 6.78 MHz. RF was applied using a shot duration of 1140 ms, with power settings ranging from 42 W to 120 W. The RF tip was applied perpendicularly to the skin surface with consistent manual pressure to ensure reproducible energy transfer. A grounding pad was placed beneath the porcine skin sample to complete the electrical circuit and ensure stable current flow during monopolar RF delivery.

#### 2.2.3. Intradermal Temperature Measurement

A calibrated microneedle-type temperature sensor (GTPK-01-40G, Giltron Inc., North Dighton, MA, USA) was used to measure intradermal temperature changes. The sensor was inserted at a consistent depth beneath the skin surface to measure intradermal temperature changes.

Temperatures were recorded at two time points: (1) immediately before RF irradiation (baseline) and (2) immediately after RF irradiation (peak temperature).

#### 2.2.4. Cumulative Heating Assessment

To investigate cumulative thermal effects, temperature measurements were performed after a single RF shot and after 10 consecutive shots at both 42 W and 73 W. This allowed comparison between single-shot and repeated-shot conditions to assess thermal effects.

### 2.3. In Vitro Study: RF Effects in Senescent Fibroblasts

This experiment aimed to investigate the effects of RF irradiation on the recovery of senescent fibroblasts.

#### 2.3.1. Cell Culture

Human dermal fibroblasts (CCD-986Sk; Korean Cell Line Bank, Seoul, Republic of Korea; no. 21947) were cultured in Iscove’s Modified Dulbecco’s Medium (Welgene, Gyeongsan, Republic of Korea) supplemented with 10% fetal bovine serum and 1% penicillin/streptomycin (growth medium). Cells were maintained at 37 °C in a humidified atmosphere containing 5% CO_2_.

#### 2.3.2. Induction of Cellular Senescence

H_2_O_2_-induced senescent cell models are widely utilized as in vitro models of cellular senescence because the oxidative stress generated by H_2_O_2_ treatment mimics senescence-associated morphological changes observed in vivo [42,43,44]. A senescent cell model was established by treating fibroblasts with H_2_O_2_; the expression levels of p16 and p21, commonly used markers of senescence, were evaluated [45].

To induce senescence, fibroblasts were exposed to 350 μM H_2_O_2_ for 1.5 h. After exposure, cells were washed with Dulbecco’s phosphate-buffered saline (DPBS; Gibco Thermo Fisher Scientific, Waltham, MA, USA) and incubated in fresh growth medium for 72 h to allow senescent phenotypes to develop [46].

#### 2.3.3. RF Irradiation and HSP70 Inhibitor Treatment

Following senescence induction, cells were treated with an HSP70 inhibitor (10 μM) for 24 h. Subsequently, RF irradiation was performed using the same RF device (Sunny; SHENB Co., Ltd.) operated at 6.78 MHz. Because a monopolar configuration requires a grounding pad and conductive tissue contact, which cannot be implemented in plastic culture dishes due to their non-conductive nature, the in vitro setup employed a bipolar electrode configuration under same energy setting (42 W, pulse duration = 1140 ms, inter-shot interval = 400 ms, 10 shots). This configuration enabled localized and uniform heating in the culture wells without electrical grounding, reproducing a thermal exposure comparable to the monopolar conditions used in animal experiments.

#### 2.3.4. Post-Treatment Incubation and Sample Collection

After RF irradiation, cells were incubated for an additional 48 h. Proteins were extracted separately from nuclear and cytosolic fractions for subsequent molecular analyses.

### 2.4. In Vivo Study: Effects of RF Irradiation on Senescent Facial Ligaments in Rats

This study aimed to assess the effects of RF irradiation on ligament recovery in UV-induced senescent facial ligaments of rats.

#### 2.4.1. Animal Model and Housing Conditions

Seven-week-old male Sprague–Dawley rats were obtained from Orient Bio (Seongnam, Republic of Korea). All animal procedures were approved by the Institutional Animal Care and Use Committee of the Lee Gil Ya Cancer and Diabetes Institute, Gachon University (Approval number: LCDI-2023-0150; Approval date, 11 November 2023), in compliance with American Association for Accreditation of Laboratory Animal Care International guidelines. Rats were housed in a temperature-controlled (20–24 °C) and humidity-controlled (45–55%) facility under a 12 h light/dark cycle, with free access to a standard diet and water available ad libitum. Animals were acclimated for 1 week before the start of the study.

#### 2.4.2. UV-Induced Senescence and RF Irradiation

A total of twenty rats were randomly assigned to four experimental groups (*n* = 5 per group) as follows: (1) Normal group (no UV exposure, no RF irradiation), (2) Senescent group (UV exposure, no RF irradiation), (3) Senescent/RF 42 W group, and (4) Senescent/RF 73 W group. These groupings were designed to compare the effects of RF energy on UV-induced senescent changes in facial ligaments. Prior to UV exposure, animals were anesthetized using isoflurane inhalation. A designated area (15 × 15 mm) on the facial skin was shaved using an electric shaver (Haseong Electronics Co., Ltd., Seoul, Republic of Korea). UV exposure was performed using a 306 nm UV lamp (Sankyo Denki Co., Ltd., Tokyo, Japan) for 1 h every other day for a total of 30 days [47]. UV radiation is a major factor that promotes cellular senescence in the skin [48]. UV-induced skin aging has been widely used as an in vivo model of senescence [49]. Accordingly, senescence in rat facial ligament was induced by repeated UV exposure following established photoaging protocols.

On day 31, RF irradiation was performed. A coupling gel (SHENB Co., Ltd.) was applied to the shaved facial skin to reduce impedance and enhance energy transfer. Under general anesthesia, animals were placed in a lateral position with the head securely fixed in place to ensure consistent RF tip placement. The RF tip was applied perpendicularly with manually maintained, uniform pressure throughout the irradiation. During RF irradiation, a standard grounding pad was attached to the animal’s abdomen to complete the monopolar circuit and maintain consistent current flow and heat distribution. To maintain consistency across experimental conditions, coupling gel application and anesthesia procedures were performed identically for all groups, including non-RF irradiated group. RF was applied using a frequency of 6.78 MHz, power of 42 W or 73 W, pulse duration of 1140 ms, an inter-shot interval of 400 ms, and a total of 10 shots.

#### 2.4.3. UV Exposure and Tissue Collection After RF Irradiation

Following RF irradiation, UV exposure was continued for an additional 20 days (1 h every other day). The total experimental period was 50 days. Two days after the final UV exposure, facial ligament tissues were collected according to a previously described protocol for histological and molecular analysis [50].

### 2.5. Sample Preparation

#### 2.5.1. RNA Extraction and cDNA Synthesis

Total RNA was extracted from fibroblasts using RNAiso Plus (TaKaRa, Tokyo, Japan), in accordance with the manufacturer’s protocol. Briefly, cells were lysed directly in RNAiso Plus reagent by adding 500 μL of RNAiso Plus to a 60 mm dish and incubating on ice for 5 min. Cells were then detached and collected using a cell scraper. Cell lysate pellets were incubated at room temperature for 5 min to allow for complete dissociation of nucleoprotein complexes. Next, chloroform (100 μL per 500 μL of RNAiso Plus) was added to the pellet; the mixture was vigorously shaken for 15 s and then incubated at room temperature for 3 min. All samples were centrifuged at 12,000× *g* for 15 min at 4 °C, resulting in three distinct phases: an upper aqueous phase containing RNA, an interphase, and a lower organic phase containing DNA and proteins. The upper aqueous phase was carefully transferred to a new RNase-free tube, and an equal volume of isopropanol was added to precipitate the RNA. The mixture was incubated at room temperature for 10 min, then centrifuged at 12,000× *g* for 10 min at 4 °C. The resulting RNA pellet was washed twice with 75% cold ethanol; this was followed by centrifugation at 7500× *g* for 5 min at 4 °C. Subsequently, ethanol was removed; the RNA pellet was air-dried for 15 min and then resuspended in RNase-free water. RNA concentration and purity were assessed using a NanoDrop spectrophotometer (Thermo Fisher Scientific) by measuring absorbance ratios at 260/280 nm and 260/230 nm. Complementary DNA (cDNA) was synthesized from 1 μg of total RNA using a reverse transcription kit (Prime-Script™ 1st Strand cDNA Synthesis Kit; TaKaRa), in accordance with the manufacturer’s instructions.

#### 2.5.2. Total Protein Isolation

Total protein was extracted from fibroblasts and ligament tissues using the EzRIPA buffer kit (ATTO Corporation, Tokyo, Japan) supplemented with a protease and phosphatase inhibitor cocktail (ATTO Corporation), in accordance with the manufacturer’s protocol. Briefly, cells and ligament tissues were washed with cold PBS and lysed in EzRIPA buffer on ice for 15 min. Cell and tissue lysates were then sonicated (high power, 10 s of sonication followed by 60 s of rest) and centrifuged at 14,000× *g* for 15 min at 4 °C. Supernatants containing total protein were collected. Protein concentrations were determined using the bicinchoninic acid assay (Thermo Fisher Scientific).

#### 2.5.3. Nuclear and Cytosolic Protein Isolation

Nuclear and cytosolic proteins were isolated using a Nuclear and Cytoplasmic Extraction Kit (Thermo Fisher Scientific), in accordance with the manufacturer’s instructions. Cells were washed with cold PBS and resuspended in cytoplasmic extraction buffer. After they had been incubated on ice for 10 min, samples were centrifuged at 12,000× *g* for 10 min at 4 °C to separate the cytoplasmic (supernatant) and nuclear (pellet) fractions. Each supernatant containing cytosolic proteins was transferred to a new tube.

The pellet containing nuclei was washed, resuspended in nuclear extraction buffer, and incubated on ice for 30 min with periodic vortexing. Each mixture was then centrifuged at 12,000× *g* for 10 min at 4 °C, and supernatants containing nuclear proteins were collected. Protein concentrations were determined using the bicinchoninic acid assay.

#### 2.5.4. Paraffin-Embedded Ligament Tissue Block

Ligament tissue was fixed in cold 4% paraformaldehyde (Sigma-Aldrich, St. Louis, MO, USA) for 72 h, then placed in a cassette and washed with distilled water. Tissues were subsequently processed using a tissue processor (Leica, Wetzlar, Germany); sequentially immersed in 95% and 99% ethanol, followed by xylene (Duksan, Ansan, Republic of Korea); and finally infiltrated with paraffin (Leica). Paraffin-embedded tissue blocks were prepared using an embedding system. Blocks were sectioned into 7 μm-thick slices using a microtome (Leica), and sections were mounted onto coated slides. Slides were incubated overnight at 60 °C to ensure proper attachment.

### 2.6. Quantitative Reverse Transcription Polymerase Chain Reaction (PCR) Detection

Quantitative PCR was conducted using SYBR Green Master Mix (TaKaRa) in total volume of 10 μL: 5 μL of SYBR Green Master Mix, 0.4 μL of forward primer, 0.4 μL of reverse primer, 1 μL of cDNA template, and 3.2 μL of nuclease-free water. The following reaction conditions were utilized: initial denaturation at 95 °C for 10 min, followed by 40 cycles of denaturation at 95 °C for 15 s, and annealing/extension at 60 °C for 1 min. Amplification was performed using a QuantStudio 5 Real-Time PCR System (Applied Biosystems, Thermo Fisher Scientific). Gene expression levels were normalized to Actb as an internal control, and relative mRNA expression levels were calculated using the 2^−ΔΔCt^ method. All experiments were conducted in triplicate to ensure reproducibility. Corresponding primer sequences are listed in Table S2.

### 2.7. Western Blot Analysis

Western blot analysis was performed to evaluate protein expression levels. Total, nuclear, and cytosolic proteins were extracted as described in Section 2.5.2 and Section 2.5.3. Equal amounts of protein (20–40 μg) were denatured by incubation with 4× lithium dodecyl sulfate buffer and 10× sample buffer (Thermo Fisher Scientific) at 70 °C for 10 min. Denatured proteins were loaded onto 10% sodium dodecyl sulfate-polyacrylamide gel electrophoresis gels and separated at 200 V for 25 min using 3-(N-morpholino) propanesulfonic acid (MOPS) buffer (Invitrogen). Proteins were subsequently transferred onto polyvinylidene difluoride membranes (Millipore, Billerica, MA, USA) using a semi-dry transfer system (ATTO) at a current of 1 A for 10 min. After transfer, membranes were blocked with 5% non-fat dry milk in Tris-buffered saline with 0.1% Tween-20 (TTBS) for 1 h at room temperature. Membranes were then incubated overnight at 4 °C with primary antibodies specific to the target proteins (Table S3). After primary antibody incubation, membranes were washed three times with TTBS and subsequently incubated with horseradish peroxidase-conjugated secondary antibodies for 1 h at room temperature. Next, membranes were extensively washed with TTBS; protein bands were detected using an enhanced chemiluminescence detection reagent (Thermo Fisher Scientific) and visualized with a ChemiDoc imaging system (Bio-Rad Laboratories, Hercules, CA, USA). Band intensities were quantified using ImageJ software 1.53s (National Institutes of Health, Bethesda, MD, USA). Relative protein expression levels were normalized to β-actin for total protein and Histone H3 for nuclear protein; values were compared with those of Non-SnCs group or Normal ligament group (first bar).

### 2.8. Sandwich ELISA Analysis

Sandwich ELISAs were performed to confirm the binding of two different antibodies. Ninety-six-well microplates were pre-coated with capture antibodies (HSP70 antibody) in 0.05 M carbonate–bicarbonate buffer (pH 9.6) and incubated overnight at 4 °C. The plates were then washed three times with phosphate-buffered saline containing 0.1% Tween-20 (TPBS) and blocked with 5% skim milk in PBS for 1 h at room temperature. After three additional washes with 0.1% TPBS, protein samples (50 μg each) were added to the wells and incubated at 4 °C for 24 h to allow antigen binding. The plates were washed again with TPBS before the addition of detection antibodies (IKKγ antibody) specific to the target protein; this step was followed by overnight incubation at 4 °C to confirm antibody binding. After plates had been washed, streptavidin–horseradish peroxidase conjugate was added to each well and incubated for 1 h at room temperature. The plates were then washed thoroughly, and substrate solution (3,3′,5,5′-tetramethylbenzidine) was added. The reaction was allowed to develop for 10–15 min prior to termination with 1 N sulfuric acid. Optical density was measured at 450 nm using a microplate reader (Thermo Fisher Scientific). Protein levels were compared with those of the non-senescent samples.

### 2.9. Immunohistochemistry

Ligament tissue sections on slides were subjected to deparaffinization and rehydration through sequential immersion in xylene, followed by graded ethanol (100–70%). After slides had been washed three times with PBS, non-specific binding was blocked by incubation with normal serum for 1 h at room temperature. Slides were then incubated with primary antibodies overnight at 4 °C (Table S3). After additional PBS wash steps, slides were incubated with biotinylated secondary antibodies (1:200; Vector Laboratories, Newark, CA, USA) for 1 h at room temperature. Slides were again washed with PBS, then incubated with an avidin-biotin complex reagent (Vector Laboratories); they subsequently were washed and incubated with 3,3′-diaminobenzidine solution (Sigma-Aldrich) for 5 min to produce a brown reaction product. Counterstaining was achieved by incubating the slides with hematoxylin (KPNT, Cheongju, Republic of Korea) for 30 s; this step was followed by washing with distilled water, dehydration, and mounting with distyrene plasticizer xylene (i.e., DPX) mounting solution (Sigma-Aldrich). Stained tissue sections were scanned using a slide scanner (Motic Scan Infinity 100; Motic, Beijing, China). Intensity measurements were analyzed using ImageJ software version 1.53s (National Institutes of Health) and compared with control samples.

### 2.10. Immunofluorescence Staining

Immunofluorescence staining was performed to visualize the localization and expression of target proteins in ligaments. After tissue slides had been subjected to deparaffinization, they were blocked with 3% normal goat serum in PBS for 1 h at room temperature to prevent non-specific binding. Slides were then incubated overnight at 4 °C with primary antibodies diluted in blocking buffer (Table S3). After primary antibody incubation, slides were washed three times with PBS and incubated with fluorophore-conjugated secondary antibodies (Alexa Fluor; Thermo Fisher Scientific) for 1 h at room temperature in the dark. Slides were again washed with PBS; nuclei were counterstained with 4′,6-diamidino-2-phenylindole (DAPI; Thermo Fisher Scientific) for 5 min. Coverslips were mounted onto glass slides using antifade mounting medium (Vector Laboratories) and sealed. Fluorescence images were acquired using a confocal laser scanning microscope (Carl Zeiss Microscopy GmbH, Jena Germany), and image analysis was performed using ZEN 2.3 software (Carl Zeiss Microscopy GmbH).

### 2.11. Masson’s Trichrome Staining

Ligament tissues were incubated in Bouin’s fluid solution (ScyTek, TRM-2; West Logan, UT, USA) for 1 h at 60 °C; rinsed three times with distilled water; and sequentially incubated in iron hematoxylin solution for 5 min, Biebrich scarlet-acid fuchsin solution for 5 min, phosphomolybdic–phosphotungstic acid solution for 12 min, and aniline blue solution for 3 min. After tissues had been stained, they were washed with distilled water, dehydrated in absolute ethanol, and mounted for microscopic observation. Fibrotic areas stained with Masson’s trichrome were quantified using ImageJ software.

### 2.12. SEM

Ligament tissue samples were fixed in Karnovsky’s fixative (2% glutaraldehyde, 2% paraformaldehyde in 0.1 M phosphate buffer, pH 7.4) for 24 h and washed twice for 20 min in 0.1 M phosphate buffer (pH 7.4). Samples were then post-fixed with 1% osmium tetroxide (OsO4) in 0.1 M phosphate buffer for 2 h and dehydrated through a graded ethanol series (50–100%). A critical point dryer (EM CPD300) was used for dehydration, and samples were coated with carbon using an ion sputter coater (EM ACE600). Ligament structures were observed using a field emission scanning electron microscope (MERLIN, Carl Zeiss Microscopy GmbH). Collagen fiber bundle diameter was analyzed using SEM images in ImageJ software (National Institutes of Health).

### 2.13. Statistical Analysis

Group comparisons were conducted using the Kruskal–Wallis test, followed by the Mann–Whitney U test for post hoc comparisons. Results are presented as the mean ± standard deviation. Statistical analyses were performed using SPSS version 26 (IBM, Armonk, NY, USA). The sample size (*n* = 5 per group) was determined according to the general recommendations for animal study [51], which suggest that a minimum of five animals per group provides sufficient statistical power to detect biologically meaningful differences while maintaining compliance with ethical standards for animal research. This sample size was therefore considered appropriate and consistent with previous studies conducted under similar experimental conditions.

## 3. Results

### 3.1. Establishing RF Application Parameters for Targeted Tissue Temperature to Increase HSP70

We first characterized the thermal and energy delivery profiles of the device. The energy fluence increased linearly with power, ranging from 10.1 J/cm^2^ at 20 W to 60.8 J/cm^2^ at 120 W. Correspondingly, the skin temperature in the ex vivo tissue increased from 2.0 °C to 29.2 °C, showing a near-linear trend with power, followed by saturation at higher levels (Table S4). The corresponding temperature elevation in ex vivo porcine skin tissue (ΔT) showed a near-linear increase to 25.5 °C at 97 W. Beyond this range, a saturation effect was observed, with temperature increases plateauing at 29.2 °C at 120 W. These findings confirmed consistent and predictable energy delivery across a broad output range (Table S4).

To further examine the cumulative thermal effects of repeated RF applications, we measured dermal temperatures following 1 and 10 consecutive RF shots at two representative power levels, 42 W and 73 W. After a single shot, the temperatures increased by 4.0 °C and 16.7 °C, following 10 consecutive shots, temperatures increased to 9.2 °C and 23.7 °C, at 42 W and 73 W, respectively (Table S5).

The fluence corresponding to these settings was 21.3 J/cm^2^ at 42 W and 37.0 J/cm^2^ at 73 W a single RF shot (Table S4). When 10 consecutive shots were applied, the cumulative fluence reached 213.0 J/cm^2^ and 370.0 J/cm^2^, respectively.

We hypothesized that HSP70 expression, induced by RF application, would mediate alterations in ligaments. To investigate whether variations in tissue temperature, resulting from RF energy, influenced HSP70 expression, we selected to apply two distinct power settings—42 W and 73 W—to the animal models. These power levels were chosen based on preliminary thermal profiling, which showed that 42 W induced intradermal temperatures around 44.8 °C—within a range reported to enhance HSP70 expression without causing thermal damage—whereas 73 W elevated temperatures to over 59 °C, a level known to elicit cellular stress and HSP70 induction [36,52]. However, for the in vitro cellular experiments, the thermal energy generated at 73 W was anticipated to be excessively high and potentially detrimental to cell viability; consequently, only the 42 W condition was applied.

### 3.2. RF-Induced Thermal Modulation Reduces NF-κB Activity via HSP70 in H_2_O_2_-Induced Senescent Fibroblasts

A senescent fibroblast model was successfully established by H_2_O_2_ treatment, as evidenced by increased expression of senescence markers p16 and p21 (Figure S1). Although the fold change in p21 expression did not exceed two-fold, the increase was statistically significant, suggesting that even a modest elevation may reflect early or partial activation of senescence signaling rather than simple physiological variation.

**Figure 1:**
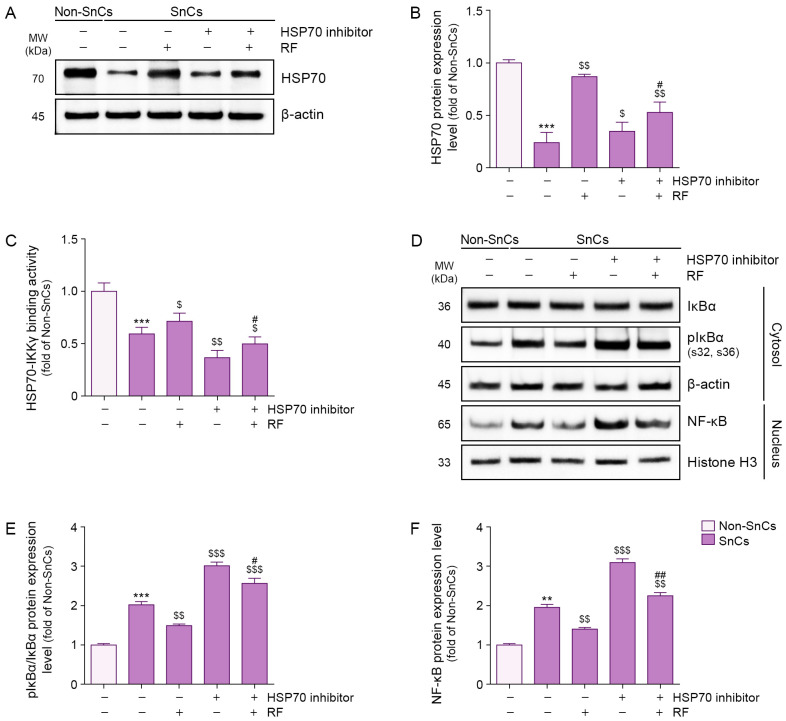
Suppression of IκBα phosphorylation and NF-κB activation via RF-mediated binding of HSP70 and IKKγ in senescent fibroblasts. Cellular senescence was induced by exposing fibroblasts to 350 μM H_2_O_2_ for 1.5 h, followed by a 72 h recovery period. Cells were then pretreated with an HSP70 inhibitor (10 μM) for 24 h prior to RF irradiation and incubated for an additional 48 h RF was applied at 42 W. (**A**) Western blot analysis of HSP70 expression in senescent fibroblasts treated with RF irradiation after HSP70 inhibitor treatment. β-actin was used as a loading control. (**B**) Quantification of HSP70 protein levels from Western blot images in panel (**A**), analyzed using ImageJ software, and normalized to β-actin, expressed as fold changes relative to the Non-SnCs group. (**C**) Binding activity between HSP70 and IKKγ in senescent fibroblasts treated with RF irradiation after HSP70 inhibitor treatment, assessed via sandwich ELISA. (**D**) Western blot analysis of IκBα and pIκBα expression in the cytosol and NF-κB expression in the nucleus of senescent fibroblasts treated with RF irradiation after HSP70 inhibitor treatment. β-actin and Histone H3 were used as loading controls for the cytosolic and nuclear fractions, respectively. (**E**,**F**) Quantification of pIκBα/IκBα (**E**) and NF-κB (**F**) protein levels from Western blot images in panel (**D**), analyzed using ImageJ software, and normalized to β-actin, expressed as fold changes relative to the Non-SnCs group. Data are presented as the mean ± standard deviation from three independent experiments. Statistical significance was determined using the Mann–Whitney U test. **, *p* < 0.01; ***, *p* < 0.001; Non-SnCs vs. SnCs. $, *p* < 0.05; $$, *p* < 0.01; $$$, *p* < 0.001; SnCs vs. all other groups. #, *p* < 0.05; ##, *p* < 0.01; SnCs/HSP70 inhibitor vs. SnCs/HSP70 inhibitor/RF. ELISA, enzyme-linked immunosorbent assay; HSP70, heat shock protein 70; IKKγ, IκBα kinase γ; MW, molecular weight; NF-κB, nuclear factor-kappa B; Non-SnCs, Non-senescent cells; pIκBα, phosphorylated IκBα; RF, radiofrequency; SnCs, senescent cells.

We evaluated the effect of RF on NF-κB activity via HSP70 in H_2_O_2_-induced senescent fibroblasts. HSP70 expression levels were lower in senescent fibroblasts than in non-senescent fibroblasts; however, RF irradiation increased these levels. Treatment of an HSP70 inhibitor reduced HSP70 expression in senescent fibroblasts, but RF irradiation restored HSP70 expression in the presence of the inhibitor (Figure 1A,B).

The interaction between HSP70 and IKKγ was assessed via sandwich enzyme-linked immunosorbent assay (ELISA). HSP70–IKKγ binding was less prominent in senescent fibroblasts than in non-senescent fibroblasts, but RF irradiation enhanced this interaction. HSP70 inhibitor treatment reduced the binding of HSP70 to IKKγ in senescent fibroblasts; however, RF irradiation restored this interaction even in the presence of the inhibitor (Figure 1C). These findings suggest that RF enhances the binding interaction between HSP70 and IKKγ in senescent fibroblasts.

Although the phosphorylated IκBα/IκBα ratio was higher in senescent fibroblasts than in non-senescent fibroblasts, RF irradiation reduced this ratio. Treatment of an HSP70 inhibitor increased the phosphorylated IκBα/IκBα ratio in senescent fibroblasts, but RF irradiation reduced this ratio even in the presence of the inhibitor (Figure 1D,E). These findings suggest that RF decreases IκBα phosphorylation via HSP70 in senescent fibroblasts.

Nuclear expression levels of NF-κB were higher in senescent fibroblasts than in non-senescent fibroblasts; however, RF irradiation reduced nuclear NF-κB expression. Whereas HSP70 inhibitor treatment increased nuclear NF-κB expression in senescent fibroblasts, RF irradiation suppressed this increase even in the presence of the inhibitor (Figure 1D,F). These findings suggest that RF reduces nuclear NF-κB expression via HSP70 in senescent fibroblasts.

### 3.3. RF-Induced Thermal Modulation Reduces MMPs and SMAD7 and Increases SMAD2/3 via HSP70 in Senescent Fibroblasts

**Figure 2:**
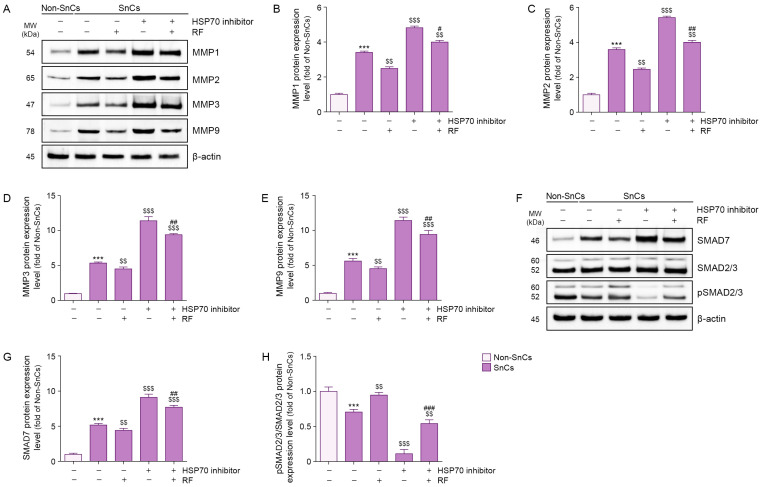
RF-mediated regulation of MMP1, MMP2, MMP3, MMP9, SMAD7, and pSMAD2/3 in senescent fibroblasts. To induce cellular senescence, fibroblasts were treated with 350 μM H_2_O_2_ for 1.5 h and allowed to recover for 72 h. Following pretreatment with HSP70 inhibitor (10 μM) for 24 h, cells were exposed to RF irradiation at 42 W and maintained in culture for an additional 48 h. (**A**) Western blot analysis of MMP1, MMP2, MMP3, and MMP9 protein expression levels in senescent fibroblasts treated with RF irradiation after HSP70 inhibitor treatment. β-actin was used as a loading control. (**B**–**E**) Quantification of protein levels of MMP1 (**B**), MMP2 (**C**), MMP3 (**D**), and MMP9 (**E**) based on Western blot data from panel A, analyzed using ImageJ software, and normalized to β-actin, expressed as fold changes relative to the Non-SnCs group. (**F**) Western blot analysis of SMAD7, SMAD2/3, and pSMAD2/3 protein expression levels in senescent fibroblasts treated with RF irradiation after HSP70 inhibitor treatment. β-actin was used as the loading control. (**G**,**H**) Quantification of protein levels of SMAD7 (**G**) and the pSMAD2/3 to SMAD2/3 ratio (**H**) based on Western blot data from panel (**F**), analyzed using ImageJ software, and normalized to β-actin, expressed as fold changes relative to the Non-SnCs group. Results are expressed as the mean ± standard deviation from three independent experiments and statistical significance was determined using the Mann–Whitney U test. ***, *p* < 0.001; Non-SnCs vs. SnCs. $$, *p* < 0.01; $$$, *p* < 0.001; SnCs vs. all other groups. #, *p* < 0.05; ##, *p* < 0.01; ###, *p* < 0.001; SnCs/HSP70 inhibitor vs. SnCs/HSP70 inhibitor/RF. HSP70, heat shock protein 70; MMP, matrix metalloproteinase; MW, molecular weight; Non-SnCs, Non-senescent cells; pSMAD2/3, phosphorylated SMAD2/3; RF, radiofrequency; SnCs, senescent cells.

Expression levels of MMP1, MMP2, MMP3, and MMP9 were higher in senescent fibroblasts than in non-senescent fibroblasts; RF irradiation reduced these levels. Treatment of an HSP70 inhibitor increased MMP1, MMP2, MMP3, and MMP9 expression levels in senescent fibroblasts, but RF irradiation suppressed these increases even in the presence of the inhibitor (Figure 2A–E). These findings suggest that RF reduces the expression levels of MMP1, MMP2, MMP3, and MMP9 via HSP70 in senescent fibroblasts.

SMAD7 expression levels were higher in senescent fibroblasts than in non-senescent fibroblasts; however, RF irradiation reduced these levels. Treatment of an HSP70 inhibitor increased SMAD7 expression in senescent fibroblasts, but RF irradiation suppressed this increase even in the presence of the inhibitor (Figure 2F,G). These findings suggest that RF reduces SMAD7 expression via HSP70 in senescent fibroblasts.

Whereas phosphorylated SMAD2/3/SMAD2/3 expression levels were lower in senescent fibroblasts than in non-senescent fibroblasts, RF irradiation increased these levels. Treatment of an HSP70 inhibitor reduced phosphorylated SMAD2/3/SMAD2/3 expression in senescent fibroblasts, but RF irradiation restored these levels even in the presence of the inhibitor (Figure 2F,H). These findings suggest that RF increases phosphorylated SMAD2/3/SMAD2/3 expression via HSP70 in senescent fibroblasts.

### 3.4. RF-Induced Thermal Modulation Reduces NF-κB Activity in Senescent Facial Ligaments

**Figure 3:**
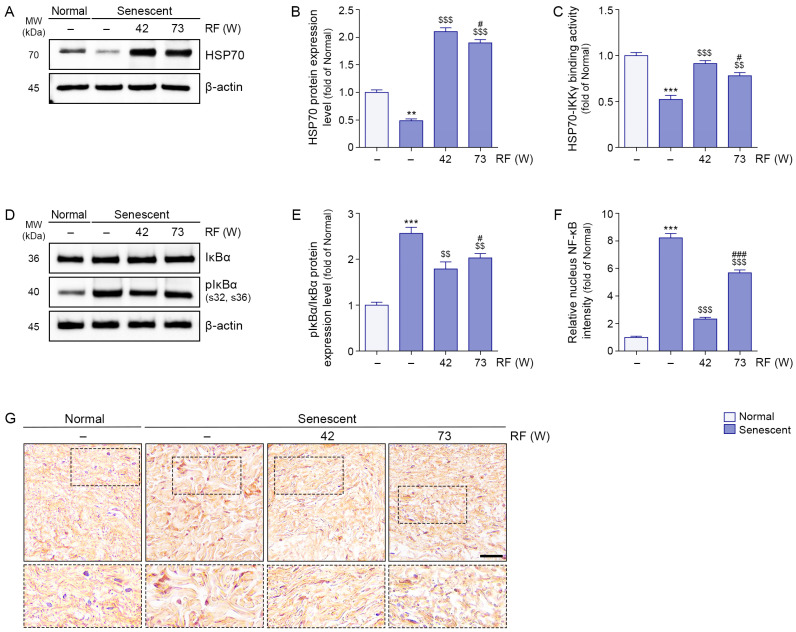
Inhibition of IκBα phosphorylation and NF-κB translocation via RF-mediated HSP70 and IKKγ binding interaction in senescent ligament tissues. RF was applied under two different energy conditions: 42 W and 73 W, with identical settings for frequency (6.78 MHz), pulse duration (1140 ms), and inter-shot interval (400 ms). (**A**) Western blot analysis of HSP70 expression levels in senescent ligament tissues after RF irradiation. β-actin was used as a loading control. (**B**) Quantification of HSP70 protein levels from Western blot data in panel (**A**), analyzed using ImageJ software, and normalized to β-actin, expressed as fold changes relative to the normal ligament group. (**C**) Binding activity between HSP70 and IKKγ in senescent ligament tissues after RF irradiation, evaluated by sandwich ELISA. (**D**) Western blot analysis of IκBα and pIκBα expression levels in senescent ligament tissues after RF irradiation. β-actin was used as a loading control. (**E**) Quantification of pIκBα/IκBα ratios from Western blot data in panel (**D**), analyzed using ImageJ software, and normalized to β-actin, expressed as fold changes relative to the normal ligament group. (**F**) Quantification of NF-κB nuclear localization based on immunohistochemistry data in panel G, analyzed using ImageJ software, and expressed as fold changes relative to the normal ligament group. (**G**) Representative immunohistochemistry images of NF-κB staining in senescent ligament tissues after RF irradiation. Dotted boxes indicate the regions that are shown at higher magnification in the lower panels. Nuclei were counterstained with hematoxylin (blue). Scale bar = 100 µm. Data are presented as the mean ± standard deviation from three independent experiments. Statistical significance was determined using the Mann–Whitney U test. **, *p* < 0.01; ***, *p* < 0.001; Normal ligament vs. Senescent ligament. $$, *p* < 0.01; $$$, *p* < 0.001; Senescent ligament vs. all other groups. #, *p* < 0.05; ###, *p* < 0.001; Senescent ligament/42 W vs. Senescent ligament/73 W. ELISA, enzyme-linked immunosorbent assay; HSP70, heat shock protein 70; IKKγ, IκBα kinase γ; MW, molecular weight; NF-κB, nuclear factor-kappa B; pIκBα, phosphorylated IκBα; RF, radiofrequency.

The induction of senescence in facial ligaments was confirmed by increased expression of the senescence markers p16 and p21 (Figure S2). Although the fold change in p21 expression did not exceed two-fold, this increase was statistically significant, suggesting that even a modest upregulation reflects biologically relevant activation of senescence signaling in ligament tissue. We induced senescence of facial ligament by UV radiation. HSP70 expression levels were lower in senescent ligaments than in normal ligaments; however, RF irradiation increased these levels. HSP70 expression was higher after RF irradiation at 42 W compared with 73 W (Figure 3A,B). HSP70–IKKγ binding was less prominent in senescent ligaments than in normal ligaments; RF irradiation enhanced this interaction. HSP70–IKKγ binding was stronger after RF irradiation at 42 W compared with 73 W (Figure 3C).

The phosphorylated IκBα/IκBα ratio was higher in senescent ligaments than in normal ligaments, but RF irradiation reduced this ratio. A greater reduction in the phosphorylated IκBα/IκBα ratio was observed after RF irradiation at 42 W relative to 73 W (Figure 3D,E). Whereas nuclear expression levels of NF-κB were higher in senescent ligaments than in normal ligaments, RF irradiation reduced these levels. A greater reduction in nuclear NF-κB expression was observed after RF irradiation at 42 W relative to 73 W (Figure 3F,G).

### 3.5. RF-Induced Thermal Modulation Reduces MMPs and SMAD7 and Increases SMAD2/3 in Senescent Facial Ligaments

**Figure 4:**
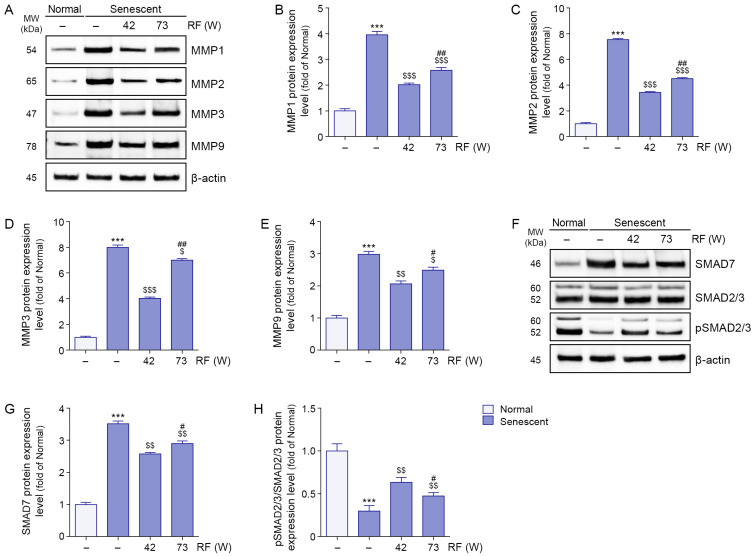
Modulation of MMPs and SMAD pathway proteins by RF irradiation in senescent ligaments. (**A**) Western blot analysis of MMP1, MMP2, MMP3, and MMP9 protein expression levels in senescent ligaments subjected to RF irradiation. β-actin was used as a loading control. (**B**–**E**) Quantification of protein levels of MMP1 (**B**), MMP2 (**C**), MMP3 (**D**), and MMP9 (**E**) based on Western blot data in panel (**A**), analyzed using ImageJ software, and normalized to β-actin, expressed as fold changes relative to the normal ligament group. (**F**) Western blot analysis of SMAD7, total SMAD2/3, and pSMAD2/3 protein expression in senescent ligaments after RF irradiation. β-actin was used as a loading control. (**G**,**H**) Quantification of SMAD7 (**G**) protein levels and the pSMAD2/3 to SMAD2/3 ratio (**H**) based on Western blot data in panel (**F**), analyzed using ImageJ software, and normalized to β-actin, expressed as fold changes relative to the normal ligament group. Data are presented as the mean ± standard deviation from three independent experiments, and statistical significance was determined using the Mann–Whitney U test. ***, *p* < 0.001; Normal ligament vs. Senescent ligament. $, *p* < 0.05; $$, *p* < 0.01; $$$, *p* < 0.001; Senescent ligament vs. all other groups. #, *p* < 0.05; ##, *p* < 0.01; Senescent ligament/42 W vs. Senescent ligament/73 W. MMP, matrix metalloproteinase; MW, molecular weight; pSMAD2/3, phosphorylated SMAD2/3; RF, radiofrequency.

Expression levels of MMP1, MMP2, MMP3, and MMP9 were higher in senescent ligaments than in normal ligaments; however, RF irradiation reduced these levels. Greater reductions in MMP1, MMP2, MMP3, and MMP9 expression levels were observed after RF irradiation at 42 W compared with 73 W (Figure 4A–E).

SMAD7 expression levels were higher in senescent ligaments than in normal ligaments, but RF irradiation reduced these levels. A greater reduction in SMAD7 expression was observed after RF irradiation at 42 W compared with 73 W (Figure 4F,G). phosphorylated SMAD2/3/SMAD2/3 expression levels were lower in senescent ligaments than in normal ligaments; RF irradiation increased these levels. Greater increases in phosphorylated SMAD2/3/SMAD2/3 expression levels were observed after RF irradiation at 42 W relative to 73 W (Figure 4F,H).

### 3.6. RF-Induced Thermal Modulation Increases Collagen Type I/III and Collagen Density in Senescent Facial Ligaments

**Figure 5:**
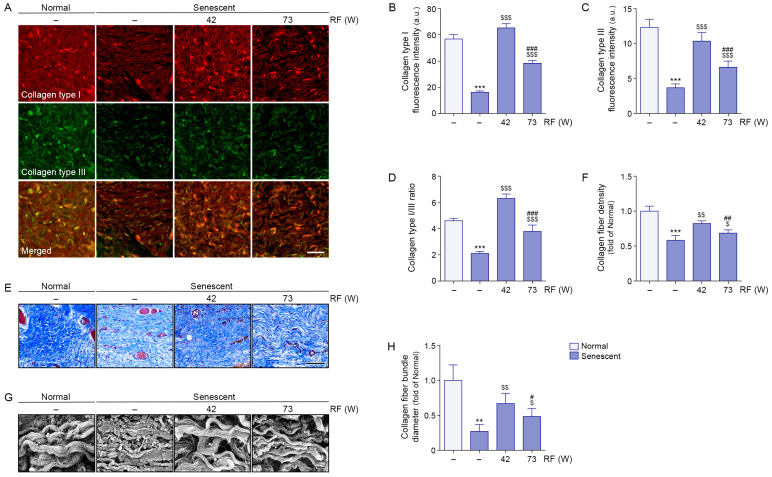
Restoration of collagen type I/III balance and fiber thickness in rat senescent ligaments by RF irradiation. (**A**) Representative immunofluorescence images showing collagen type I (red) and collagen type III (green) in senescent ligaments treated with RF irradiation. Scale bar = 50 µm. (**B**,**C**) Quantification of fluorescence intensity for collagen type I (**B**) and collagen type III (**C**) based on immunofluorescence data in panel (**A**), analyzed using ZEN microscopy software. (**D**) The collagen type I/III ratio was calculated using ZEN microscopy software. (**E**) Representative Masson’s trichrome staining images showing collagen fiber density in senescent ligaments after RF irradiation. Collagen fibers are stained blue. Scale bar = 100 µm. (**F**) Quantification of collagen fiber density based on Masson’s trichrome staining in panel E, analyzed using ImageJ software. (**G**) Representative SEM images of ligament fibers in senescent ligaments after RF irradiation. (**H**) Measurement of collagen fiber bundle diameter using ImageJ software, based on SEM images in panel (**G**), with values expressed as fold changes relative to the normal ligament group. Data are presented as the mean ± standard deviation from three independent experiments. Statistical significance was determined using the Mann–Whitney U test. **, *p* < 0.01; ***, *p* < 0.001; Normal ligament vs. Senescent ligament. $, *p* < 0.05; $$, *p* < 0.01; $$$, *p* < 0.001; Senescent ligament vs. all other groups. #, *p* < 0.05; ##, *p* < 0.01; ###, *p* < 0.001; Senescent ligament/42 W vs. Senescent ligament/73 W. DAPI, 4′,6-diamidino-2-phenylindole; RF, radiofrequency; SEM, scanning electron microscopy.

Collagen type I/III expression levels were lower in senescent ligaments than in normal ligaments; RF irradiation increased these levels. Greater increases in collagen type I/III expression levels were observed after RF irradiation at 42 W compared with 73 W (Figure 5A–C). The collagen type I/III ratio was lower in senescent ligaments than in normal ligaments; however, RF irradiation increased this ratio. A greater increase in the collagen type I/III ratio was observed after RF irradiation at 42 W compared with 73 W (Figure 5D).

Collagen fiber density in ligaments was evaluated using Masson’s trichrome staining. Although collagen fiber density was lower in senescent ligaments than in normal ligaments, RF irradiation increased collagen fiber density. A greater increase in collagen fiber density was observed after RF irradiation at 42 W relative to 73 W (Figure 5E,F).

In the present study, SEM imaging revealed that the diameter of collagen fiber bundles was smaller in senescent ligaments than in normal ligaments; however, RF irradiation increased the fiber bundle diameter. A greater increase in collagen fiber density was observed after RF irradiation at 42 W relative to 73 W (Figure 5G,H).

In senescent ligaments, fiber bundles appeared fragmented and exhibited a loss of helical structure. RF irradiation reduced fiber fragmentation and restored the helical configuration of collagen fibers in the ligament.

## 4. Discussion

The human face comprises multiple layers, including skin, subcutaneous adipose tissue, muscle, deep adipose layer, ligaments, and bones [53]. All facial layers change during aging. Skin wrinkling, sagging, and facial volume loss require appropriate corrective techniques to address skin laxity, restore fat volume, and reposition soft tissue [54,55]. During aging, gravitational forces contribute to soft tissue descent [2]. Additionally, ligament weakening promotes the exertion of medial and downward vectors. The sum of these vectors generates a primary diagonal downward force, resulting in soft tissue displacement [2]. Therefore, ligament weakening may contribute to both skin sagging and laxity [2,3].

Energy-based nonsurgical modalities, including laser therapy, RF, and focused ultrasound, have been utilized for skin tightening through the induction of thermal collagen contraction and ECM remodeling [19]. In addition to their effects on the dermis, energy-based nonsurgical modalities influence collagen-containing structures such as the superficial muscular aponeurotic system [1]. RF promotes collagen synthesis by upregulating HSPs [26]. RF irradiation also increases the expression of HSP47, HSP90, and TGF-β in H_2_O_2_-induced senescent keratinocytes [56]. Moreover, RF changed skin transcriptome [57]. RF led to transcriptional upregulation of FOS and downregulation of MMP9, which was related to changes in the macrophage activation and fibroblasts [57].

Although RF-mediated collagen accumulation in the dermis is well documented, and facial ligaments consist of collagen fibers, the effects of RF on collagen fibers within facial ligaments have not yet been investigated. This study explored whether RF induces collagen changes in facial ligaments using both in vitro and in vivo senescence models.

Non-lethal stressors induce cellular senescence, a process known as stress-induced premature senescence (SIPS) [42,58]. In vitro models of SIPS can be generated by exposing cells to environmental stress factors [42,58]. Established SIPS inducers include mitomycin C, other cytostatic drugs, H_2_O_2_, tert-butyl hydroperoxide, copper sulfate, diperoxovanadate, ethanol, and UV radiation [42,59,60,61,62]. Exposure of proliferative cells to these SIPS inducers replicates cellular senescence, thereby mimicking oxidative stress-associated aging processes [42,59,60,61,62].

The present study required facial ligaments near the zygomatic bone for analysis. Proper separation of facial ligaments was not feasible in mice because of their small size; therefore, rats were used. UV-induced photoaging, commonly utilized in skin rejuvenation research, is associated with increased expression levels of senescence markers such as p21 and p16 [63,64]. Accordingly, we generated senescent ligaments by exposing rats to UV radiation.

For the senescent fibroblast model, we used H_2_O_2_ instead of UV radiation, which mainly affects keratinocytes and subsequently influences other dermal cells (e.g., melanocytes and fibroblasts) [65]. Ligaments and tendons consist of fibroblast-like cells, such as tenocytes and ligament fibroblasts, embedded within an ECM [66,67]. These fibroblast-like cells are responsible for collagen fiber synthesis in ligaments and tendons [66,67]. This study was conducted to determine whether RF influences senescent facial ligaments and induces changes in collagen fibers. Therefore, we regarded senescent fibroblasts as an appropriate in vitro model to evaluate RF effects on ligaments.

Given that keratinocytes are the primary targets of UV radiation and subsequently influence fibroblasts, an alternative approach would involve irradiating keratinocytes with UV and then treating fibroblasts with conditioned media derived from the irradiated keratinocytes. However, the use of conditioned media would introduce additional complexity to the experimental design. To maintain simplicity, we selected an H_2_O_2_-induced senescent model, in which fibroblasts were directly treated with H_2_O_2_. Expression levels of p21 and p16 were evaluated after H_2_O_2_ treatment. Increased expression levels of p21 and p16 were observed, indicating that the H_2_O_2_-induced senescent fibroblast model was suitable for testing the study hypothesis.

Next, we evaluated the expression levels of HSP70 and NF-κB in senescent fibroblasts. HSP70 expression and subsequent binding to IKKγ were decreased in senescent fibroblasts but increased by RF irradiation. IκBα phosphorylation was enhanced in senescent fibroblasts and reduced after RF irradiation. The effects of senescence on HSP70 expression, HSP70–IKKγ binding, and IκBα phosphorylation were abolished by HSP70 inhibitor treatment. These findings suggest that HSP70 regulates HSP70–IKKγ binding and IκBα phosphorylation during senescence. After RF irradiation in fibroblasts treated with an HSP70 inhibitor, HSP70–IKKγ binding increased, whereas IκBα phosphorylation decreased. These findings indicate that RF modulates HSP70 to regulate HSP70–IKKγ binding and IκBα phosphorylation.

Both the increased binding of HSP70 to IKKγ and the decreased phosphorylation of IκBα contribute to diminished NF-κB activation [29,30,31]. NF-κB activity, assessed through nuclear NF-κB expression, was elevated in senescent fibroblasts but exhibited reduction after RF irradiation. This RF-mediated reduction in NF-κB activity was attenuated by HSP70 inhibitor treatment, suggesting that RF decreases NF-κB activity by modulating HSP70 in senescent fibroblasts.

NF-κB activity influences both collagen degradation and synthesis [9,33,34,35]. Expression levels of MMP1, MMP2, MMP3, and MMP9 were enhanced in senescent fibroblasts but showed a decline after RF irradiation. Additionally, senescence increased SMAD7 expression and reduced SMAD2/3 expression, whereas RF irradiation reversed these effects. The RF-mediated changes in expression levels of MMP1, MMP2, MMP3, MMP9, SMAD7, and SMAD2/3 were mitigated by HSP70 inhibitor treatment. These findings suggest that RF increases HSP70 expression; decreases NF-κB activation via HSP70 modulation; and subsequently affects the expression levels of MMP1, MMP2, MMP3, MMP9, SMAD7, and SMAD2/3.

Energy-based nonsurgical devices generate heat, which increases tissue temperature. The extent of tissue response varies according to temperature and exposure time [36]. At 40–45 °C, enzymes and membranes are denatured [68]. At 60 °C, tissue necrosis and protein denaturation are induced [68]. Collagen fiber contraction occurs at 57–61 °C [20,69,70]; however, the extent of contraction varies by temperature and exposure time [71]. At 85 °C, collagen undergoes structural changes with only 1 ms of exposure [72], whereas at 67 °C, structural changes require 3 ms of exposure [72]. Collagen fiber remodeling is safer when induced at a lower temperature of 43 °C with an exposure duration of 3–5 min [72].

In our animal models, RF was applied at two energy conditions to evaluate its effect on HSP70 in an energy intensity-dependent manner. HSP70 expression was decreased in senescent ligaments but increased after RF irradiation. HSP70–IKKγ binding was also diminished in senescent ligaments but showed enhancement upon RF irradiation. IκBα phosphorylation and NF-κB activity were elevated in senescent ligaments and reduced by RF irradiation. The expression levels of MMPs and SMAD7 were increased in senescent ligaments but decreased after RF irradiation. In contrast, SMAD2/3 phosphorylation was reduced in senescent ligaments; they were restored via RF irradiation. Collagen type I/III expression levels, as well as the collagen type I/III ratio, were decreased in senescent ligaments but increased upon RF irradiation. Additionally, collagen fiber bundle diameter showed a decline in senescent ligaments; this change was mitigated by RF irradiation.

Previous studies showed collagen fibers within the tendons of young humans exhibit a more pronounced helical pattern compared with the tendons of older individuals [73]. In contrast, the tendons of older individuals display fragmented and disorganized collagen fibrils with a reduced helical structure, as observed in SEM images [73]. Our findings indicate that RF irradiation preserved collagen fiber integrity and helical structure in aged ligaments, contrasting with the severe disruption observed without treatment. These findings suggest that RF can restore the collagen type I/III ratio and fiber bundle diameter and structure in senescent ligaments; the changes were more pronounced when RF was applied at 42 W, rather than 73 W.

This study did not determine the exact mechanism by which RF mitigated senescence-related changes in the ligament more effectively when applied at 42 W compared with 73 W. However, such changes may be attributed to differences in HSP70 expression depending on energy conditions. Considering that HSP70 expression was higher at 42 W than at 73 W, downstream signaling pathways regulated by HSP70 presumably were more strongly influenced at 42 W. The different expression of HSP70 between 42 W and 73 W could be induced by local tissue temperature increase by RF. The exact mechanism regarding how the RF energy condition affects the expression of HSP70 should be evaluated in the future study. Since we assumed that RF increases HSP70 expression by generating heat in the tissue, we did not investigate changes in HSP70 expression following RF exposure within a non-thermal range. However, as there may be other physical stimuli besides heat that can upregulate HSP70, future studies should examine whether RF can increase HSP70 expression independently of heat generation.

RF induces collagen fiber contraction and promotes collagen production, making it a widely used modality for skin rejuvenation. Additionally, RF has been utilized to facilitate the regeneration of ligaments and tendons, which are primarily composed of collagen fibers. In the context of anterior cruciate ligament repair, RF-mediated shrinkage of the anterior cruciate ligament reduces ligament laxity, promoting postoperative functional recovery [74]. An animal model study showed that RF can also enhance the wound-healing process in the patellar ligament by increasing TGF-β expression [23].

Although facial ligament laxity contributes to the descent of facial structures over the bone, the effects of RF on facial ligaments have not been previously investigated. The present findings suggest that RF attenuates senescence-related changes in facial ligaments by modulating HSP70. However, we did not evaluate whether these ligament changes result in the restoration of a youthful facial shape in humans. Clinical studies are needed to explore this effect. In this study, the term “preservation or enhancement of ligament structure” refers to histological and molecular changes—such as collagen fiber remodeling and HSP70 modulation–rather than mechanical strengthening or clinical lifting effects. Therefore, our findings should be interpreted as preclinical evidence supporting molecular stabilization of ligament collagen fibers within ligaments, rather than direct proof of structural reinforcement in vivo. If RF reduces facial ligament laxity and restores a more youthful facial structure, it may serve as a novel non-invasive modality for correcting facial soft tissue descent. Considering the large size of the RF device tip we used, it is not possible to conclude that RF energy was selectively applied only to the rat’s facial ligaments. Therefore, based solely on these experimental results, we cannot differentiate whether RF directly impacted the ligaments or if changes in surrounding tissues secondarily led to alterations in the ligaments. Future studies should utilize larger animal models with more selective RF applications to further evaluate ligament-specific changes. We acknowledge this limitation and the broader absence of functional or clinical correlation, such as ligament mechanical strength or aesthetic evaluation, which is critical for validating clinical relevance. Future studies employing larger animal models with more precise RF applications are necessary to clarify ligament-specific effects and their translation to clinical benefits.

While the present study provides preclinical evidence that low-energy radiofrequency (42 W) more effectively induces molecular rejuvenation pathways via HSP70 than higher energies (73 W) in senescent animal ligaments, translation to human clinical settings must carefully consider tissue thickness variation and individual anatomy. Achieving the optimal subsurface temperature (typically 45–60 °C for collagen remodeling) depends on factors such as overlying skin, subcutaneous fat, and patient morphotype, as thicker tissues or greater adiposity can alter heat dispersion and required energy [75,76,77]. Ensuring epidermal temperatures remain below 42–45 °C is critical for safety [78,79]. Advanced approaches, such as temperature feedback or imaging-guided RF [80], could help accurately titrate energy delivery in vivo and personalize protocols. Future studies should investigate real-time temperature mapping and develop predictive models to optimize RF-induced rejuvenation across varying anatomic sites and patient morphologies. Additionally, investigating the long-term effects and conducting a detailed comparison of energy-dependent responses between 42 W and 73 W settings will be important to fully understand and optimize therapeutic outcomes.

Moreover, we did not evaluate long-term complication of RF in this study. However, complication rates associated with monopolar RF are reported very low in previous studies [81,82]. For example, the incidence of second-degree burn was 0.36% [81], and tissue irregularity due to overheating occurred in 0.08% of 151,000 uses [82]. In addition to these rare events, other potential adverse effects have been reported, including altered sensation, erythema, blistering, crusting, scarring, hypopigmentation, urticaria (itching), and reactivation of herpes simplex virus. Even though a low complication rate was expected based on previous studies, long-term complication should be further evaluated in the human study for using RF on facial ligament.

## 5. Conclusions

**Figure 6:**
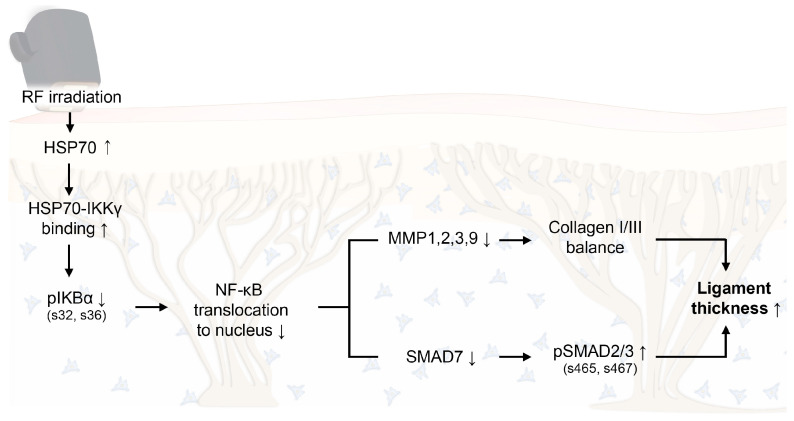
Proposed schematic illustration of the molecular mechanism by which RF-induced thermal modulation alleviates senescence-related degradation of collagen in facial ligaments. RF upregulates HSP70 expression, promotes HSP70–IKKγ binding, and reduces IκBα phosphorylation, thereby decreasing NF-κB translocation to the nucleus. Consequently, MMP1/2/3/9 and SMAD7 are downregulated, while pSMAD2/3 activation and collagen I/III balance are enhanced, resulting in increased collagen fiber bundle diameter in facial ligaments. HSP70, heat shock protein 70; IKKγ, IκBα kinase γ; MMP, matrix metalloproteinase; NF-κB, nuclear factor-kappa B; pIκBα, phosphorylated IκBα; RF, radiofrequency.

This study demonstrated that RF increased HSP70 expression, which enhanced HSP70–IKKγ binding and reduced IκBα phosphorylation in cultured senescent fibroblasts and the senescent facial ligaments of animal models. These changes decreased NF-κB activity and downregulated its downstream targets, including MMPs and SMAD7, in senescent fibroblasts and the senescent facial ligaments of animal models. Consequently, RF irradiation restored the collagen type I/III ratio and increased collagen fiber bundle diameter in facial ligaments (Figure 6). Because facial ligament laxity contributes to soft tissue descent, the modulation of facial ligaments should be considered a potential strategy for restoring a youthful facial structure. RF irradiation demonstrated the potential to mitigate senescence-related changes in facial ligaments.

## Data Availability

All data are contained within the article.

## Supplementary Materials

The following supporting information can be downloaded from https://www.mdpi.com/article/10.3390/cells14221757/s1Figure S1: Expression of p16 and p21 markers in senescent fibroblasts; Figure S2: Expression of p16 and p21 markers in senescent ligaments; Table S1: Overview of experimental designs for RF irradiation studies; Table S2: List of primers for quantitative polymerase chain reaction; Table S3: List of antibodies used for Western blot, ELISA, DAB and IF; Table S4: Changes in fluence and temperature (ΔT) according to power output; Table S5: Dermal temperature changes after 1 shot and 10 shots RF irradiation at 42 W and 73 W.
