# Supplementary material for: Radiofrequency-Induced Thermal Modulation Reduces Senescence-Induced Collagen Fiber Degradation in Facial Ligaments of Animal Models
Source: Cells. 2025 Nov 10;14(22):1757. doi: 10.3390/cells14221757 (PMC12651331; doi:10.3390/cells14221757)
Supplement: Supplementary file 1 [file cells-14-01757-s001.zip › cells-3938561-supplementary.pdf]

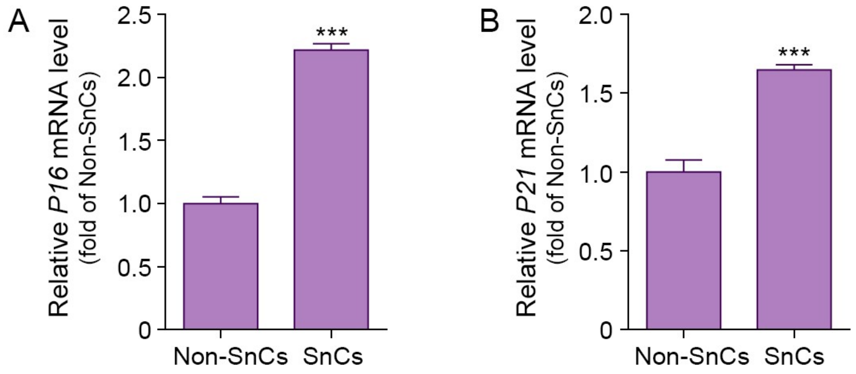

Figure S1. Expression of p16 and p21 markers in senescent fibroblasts. (**A**,**B**) mRNA expression levels of senescence markers p16 (A) and p21 (B) were measured in non-senescent fibroblasts (Non-SnCs) and H_2O_2-induced senescent fibroblasts (SnCs). Expression levels were quantified via qPCR, and data are presented as the mean ± standard deviation from three independent experiments. Statistical significance was determined using the Mann–Whitney U test. ***, $p < 0.001$, Non-SnCs vs. SnCs. Non-SnCs, Non-senescent cells; SnCs, senescent cells; qPCR, quantitative reverse transcription polymerase chain reaction assays.

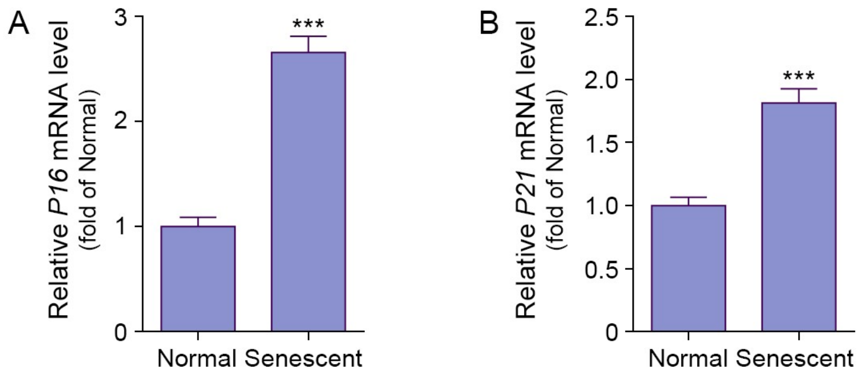

Figure S2. Expression of p16 and p21 markers in senescent ligaments. (**A, B**) mRNA expression levels of the senescence markers p16 (A) and p21 (B) were measured by qPCR in normal and senescent ligaments. Data are presented as the mean ± standard deviation from three independent experiments. Statistical significance was determined using the Mann–Whitney U test. ***, $p < 0.001$, Normal vs. Senescent. qPCR, quantitative reverse transcription polymerase chain reaction assays.

Table S1. Overview of experimental designs, objectives, and endpoints for RF irradiation studies

	Ex vivo study	In vitro study	In vivo study
**Objective**	To evaluate thermal changes in porcine skin induced by RF irradiation	To investigate the effects of RF irradiation in senescent fibroblasts	To assess the effects of RF irradiation on ligament recovery in UV-induced senescent facial ligaments
**Primary endpoint**	Peak intradermal temperature (°C) in porcine skin after RF irradiation	Molecular analysis of ligament recovery in senescent fibroblasts post-RF irradiation	Histological and molecular analysis of ligament recovery after RF irradiation in UV-induced senescent facial ligaments
**Secondary endpoint**	Comparison of temperature elevation between single and 10-shot RF application at 42 W and 73 W	–	Comparative effect of RF power (42 W vs. 73 W)
**Study design**	• Sample: Excised porcine skin (uniform thickness and size)<br>• Preconditioning: Vacuum-sealed, preheated to 35.5–37.0°C<br>• Intervention: RF applied at 42–120 W, 10 shots per application	• Cell line: Human dermal fibroblasts (CCD-986Sk)<br>• Induction of Senescence: H_2O_2 exposure (350 μM, 1.5 hour) → 72 hr recovery in fresh medium<br>• Intervention: HSP70 inhibitor (10 μM, 24 hour) treatment then RF (42 W, 10 shots) irradiation	• Animals: Male Sprague–Dawley rats (n=20, 4 groups)<br>• UV-senescence induction: UV exposure (1 hour every other day for 30 days)<br>• Intervention: RF 42 W or 73 W (10 shots) applied to facial area post-UV exposure
**Measurement process**	• Instrumentation: Calibrated microneedle-type temperature sensor (GTPK-01-40G)<br>• Time points: Temperature measured pre- and post-RF application<br>• Cumulative Assessment: Single vs. 10 consecutive shots (at 42 W, 73 W)	• Sampling: Protein extraction from nuclear & cytosolic fractions at 48 h post-RF<br>• Assays: Molecular analyses (e.g., Western blot, ELISA—details in full manuscript)	• Sampling: Facial ligaments collected 20 days after RF irradiation<br>• Assays: Histological and molecular analyses (For histological analyses: IHC, Masson trichrome staining, SEM/ For molecular analyses: Western blot, ELISA-details in full manuscript)

Table S2. List of primers for quantitative polymerase chain reaction.

Gene *(Organism)*	Primer sequences	
ACTB (human)	Forward	5'-GGG ACC TGA CTG ACT ACC TCA T-3′
	Reverse	5'-CCT TAA TGT CAC GCA CGA TTT-3′
P16 (human)	Forward	5'-GGC CTT CTT CCT CTT CTG CT-3′
	Reverse	5'-GCA ACA TCA CCA ATG GAC AG-3′
P21 (human)	Forward	5'-ATA TCA GGA AAA AGG GTG CAG-3′
	Reverse	5'-CAG AAT GAG GAA CTC CTG GAA G-3′
Actb (rat)	Forward	5'-GGG ACC TGA CTG ACT ACC TCA T-3′
	Reverse	5'-CCT TAA TGT CAC GCA CGA TTT-3′
p16 (rat)	Forward	5'-GGC CTT CTT CCT CTT CTG CT-3′
	Reverse	5'-GCA ACA TCA CCA ATG GAC AG-3′
p21 (rat)	Forward	5'-ATA TCA GGA AAA AGG GTG CAG-3′
	Reverse	5'-CAG AAT GAG GAA CTC CTG GAA G-3′

Table S3. List of antibodies used for western blot, ELISA, DAB and IF.

Antibody	Dilution rate			
	Western blot	ELISA	DAB	IF
β-actin	1:1,000			
Histone H3	1:1,000			
HSP70	1:500	1:200		
IκBα	1:1,000			
IKKγ		1:100		
MMP1	1:1,000			
MMP2	1:1,000			
MMP3	1:1,000			
MMP9	1:1,000			
NF-κB	1:500		1:200	
pIκBα	1:1,000			
pSMAD2/3	1:1,000			
SMAD7	1:1,000			
SMAD2/3	1:1,000			
Collagn type I				1:100
Collagen type III				1:100

The table presents the list of antibodies used in this study for Western blot, ELISA, DAB and IF. DAB, 3,3′-diaminobenzidine; ELISA, Enzyme-linked immunosorbent assay; IF, immunofluorescence; IKKγ, IκBα kinase γ; MMP, matrix metalloproteinases; NF-κB, nuclear factor-kappa B; pIκBα, phosphorylated IκBα; pSMAD2/3, phosphorylated SMAD2/3.

Table S4. Changes in fluence and temperature (ΔT) according to power output.

Power (W)	Fluence (J/cm²)	Δ T (°C)
20	10.1	2.0
27	13.7	3.1
35	17.7	3.7
42	21.3	4.0
50	25.3	6.1
57	28.9	10.0
65	32.9	14.3
73	37.0	16.7
81	41.0	19.7
89	45.1	22.0
97	49.1	25.5
105	53.2	26.2
113	57.3	27.0
117	59.3	27.4
119	60.3	28.6
120	60.8	29.2

RF energy was applied using a fixed frequency of 6.78 MHz, and a pulse duration of 1,140 ms. The table presents calculated fluence (J/cm²) and measured intradermal temperature increases (ΔT, °C) in porcine skin after a single RF shot at various power settings (20–120 W). Fluence values increased linearly with power, and the corresponding temperature elevation showed a consistent upward trend, confirming the predictable thermal response of the RF system. MHz, megahertz; ms, millisecond; RF, radiofrequency; W, watt.

Table S5. Dermal temperature changes after 1 shot and 10 shots RF irradiation at 42 W and 73 W.

**Power (W)**	**Baseline Temperature (°C)**	**Peak Temperature (°C) after RF irradiation**	
		**1 shot**	**10 shots**
42	35.65 ± 0.40	39.65 ± 1.02	44.81 ± 1.70
73	35.55 ± 0.39	52.25 ± 2.02	59.27 ± 2.96

Skin temperature was measured in porcine tissue following RF irradiation at 42W and 73W. Baseline temperature was recorded immediately before irradiation, and peak temperature was measured immediately after 1 or 10 consecutive RF shots. All conditions shared the same frequency (6.78 MHz) and pulse duration (1,140 ms). Data are presented as the mean ± standard deviation from three independent experiments. MHz, megahertz; ms, millisecond; RF, radiofrequency; W, watt.
